# Misconduct Policies, Academic Culture and Career Stage, Not Gender or Pressures to Publish, Affect Scientific Integrity

**DOI:** 10.1371/journal.pone.0127556

**Published:** 2015-06-17

**Authors:** Daniele Fanelli, Rodrigo Costas, Vincent Larivière

**Affiliations:** 1 Meta-Research Innovation Center at Stanford (METRICS), 1070 Arastradero Road, Stanford University, Palo Alto, 94304, California, United States of America; 2 Center for Science and Technology Studies, Leiden University, Wassenaarseweg 62A, 2333 AL, Leiden, The Netherlands; 3 École de bibliothéconomie et des sciences de l'information, Université de Montréal, C.P. 6128, Succ. Centre-Ville, Montréal, QC, H3C 3J7, Canada, and OST-CIRST, Université du Québec à Montréal, C.P. 8888, Succ. Centre-Ville, Montréal, QC, H3C 3P8, Canada; State University of New York, Oswego, UNITED STATES

## Abstract

The honesty and integrity of scientists is widely believed to be threatened by pressures to publish, unsupportive research environments, and other structural, sociological and psychological factors. Belief in the importance of these factors has inspired major policy initiatives, but evidence to support them is either non-existent or derived from self-reports and other sources that have known limitations. We used a retrospective study design to verify whether risk factors for scientific misconduct could predict the occurrence of retractions, which are usually the consequence of research misconduct, or corrections, which are honest rectifications of minor mistakes. Bibliographic and personal information were collected on all co-authors of papers that have been retracted or corrected in 2010-2011 (N=611 and N=2226 papers, respectively) and authors of control papers matched by journal and issue (N=1181 and N=4285 papers, respectively), and were analysed with conditional logistic regression. Results, which avoided several limitations of past studies and are robust to different sampling strategies, support the notion that scientific misconduct is more likely in countries that lack research integrity policies, in countries where individual publication performance is rewarded with cash, in cultures and situations were mutual criticism is hampered, and in the earliest phases of a researcher’s career. The hypothesis that males might be prone to scientific misconduct was not supported, and the widespread belief that pressures to publish are a major driver of misconduct was largely contradicted: high-impact and productive researchers, and those working in countries in which pressures to publish are believed to be higher, are less-likely to produce retracted papers, and more likely to correct them. Efforts to reduce and prevent misconduct, therefore, might be most effective if focused on promoting research integrity policies, improving mentoring and training, and encouraging transparent communication amongst researchers.

## Introduction

The honesty and integrity of scientists is commonly assumed to depend on multiple structural, sociological, and psychological factors [1]. The factors most commonly discussed in the literature include:

1. **Policies**: the presence, at the institutional or national level, of policies and structures to detect and punish misbehaviour and to promote research integrity is assumed to bolster scientific self-correction and deter scientists from engaging in irresponsible behaviour [2–4].
2. **Culture**: socio-cultural background is believed to play a role in research misconduct [5]. A particularly elaborate and testable theory was proposed by Lee and Schrank 2010 [6], who argued that the risk of scientific misconduct would be highest in “developmental” states (i.e. countries in which economic growth is prioritized at the expense of regulation) that adopted a German model of higher education, which is more hierarchical and less liberal. The risk should instead be lowest in “regulatory” states that adopted an Anglo-American model of higher education, because in these countries researchers are held accountable for their actions and mutual criticism takes precedence over respect for authority. China, South Korea and other countries would be examples of the former category, the UK and USA of the latter, whilst Germany (which is a regulatory state) and other countries would represent intermediate cases [6].
3. **Peer control**: at the core of the scientific method lies the principle that transparent, open communication and mutual criticism are essential elements of scientific self-correction. Scholars have therefore suggested that, when such elements are missing, for example when mentorship of trainees is inadequate and/or when collaborators are unable or discouraged from checking and criticising each others’ work, fraudulent or questionable behaviours become more likely [7].
4. **Pressures to publish**: in most scientifically active countries, competition for jobs and resources is growing and career success is determined to some extent by research performance. This puts virtually all scientists under some “pressures to publish” [8]. However, in several countries, productivity and impact are formally built into promotion criteria, which may force scientists to publish continuously and successfully to maintain their careers [9]. In a growing number of countries, moreover, institutions receive funding in proportion to their publication performance and might therefore make explicit pressures on their employees [10,11]. Finally, in a few countries researchers are rewarded with cash incentives, which are arguably not the source of actual pressures but rather a source of corruption [12].
5. **Early-career**: young researchers are believed to be particularly at risk of committing scientific misconduct for at least two interconnected reasons. On the one hand, they may not have fully internalized the ethos and principles of science [13]. On the other hand, they have not established a professional reputation yet, so they may have more to gain and less to lose from attempting fraud [14].
6. **Gender**: at least two independent studies suggested that males were overrepresented in findings of misconduct by the US Office of Research Integrity. This supported speculations that psychological characteristics including higher aggression, competitiveness, status-seeking and risk-taking, made males a high-risk category for scientific misconduct [15,16]. Other interpretations, however, are at least as plausible [17].

A firm belief in the importance of some of these factors underlies major policies and educational initiatives. Major research institutions, for example in Germany and The Netherlands, have revised their research evaluation criteria, following the unquestioned concern that productivity expectations are a major threat to scientific integrity [18,19]. Institutions around the world are being encouraged to establish regulations and structures to deal with cases of misconduct, under the assumption that such structures will have beneficial effects [3]. In the United States and other countries, universities are required to implement training in the responsible conduct of research, following the belief that early-career scientists are most susceptible to misbehaviour and need specific instruction [7,14].

What is the evidence to support such policies and their underlying beliefs? The most explicit connections between scientific misconduct and pressures to publish or other risk factors comes from surveys and focus groups [e.g. 9,10,13,16,20,21], sources that have known limitations. What scientists report when interviewed is of great use in many contexts, but ultimately reflects personal impressions and beliefs, and might not directly echo what scientists actually do. Moreover, results of surveys on research misconduct are significantly influenced by methodological choices and publication bias [22]. Less indirect evidence about scientific misconduct comes from studies that surveyed the literature using proxies of publication bias. These suggest that the ratio of positive to negative results is unequally distributed amongst disciplines [23], is growing in most fields and countries [24,25], and is higher in long-distance collaborations [26] and in scientifically productive countries [27–29]. These patterns lend independent support to conclusions derived by survey data about the prevalence of pressures to publish and other risk factors. However, studies measuring publication bias cannot separate the effects of actual scientific misconduct from those of conscious or unconscious biases, editorial decisions or even factors that are completely unrelated to scientific integrity, such as writing style [22,30].

A more promising source of evidence for scientific misconduct is offered by retractions of scientific papers, because these are usually the consequence of data fabrication, falsification and plagiarism [31]. Existing studies on retractions, however, have failed to control for important confounding factors, thus yielding inconclusive results. Analyses of retraction notices recorded in Medline have led researchers to suggest that scientific misconduct is growing and is particularly common in high-impact journals [32,33] and that data falsification might be more common in long distance collaborations [34]. These conclusions might be misguided, however, because they ignored the effects of policies, structures and cultures in which researchers operate—factors that might vary significantly over time and across countries, institutions, research fields, and journals [35]. The recent growth in retractions, for example, is entirely accounted for by the number of journals that have started to retract papers, so it is not a sign that scientific misconduct has increased [35]. Another major confounding factor in studies on retractions is represented by “prolific retractors”: uncovered cases of misconduct are increasingly likely to yield multiple retractions, and ignoring this fact might have skewed results of past analyses [36].

In addition to retractions, a powerful source of evidence about scientific integrity—and one that has been surprisingly overlooked by scholars—is represented by corrections to the scientific literature. Unlike retractions, corrections carry no stigma and do not affect the publication record, so they have no direct consequence on a scientist’s career. Unlike retractions, which are often accompanied by litigations and lengthy investigations (for current examples see retractionwatch.com), corrections are typically a friendly process, often solicited spontaneously by the authors of the erroneous paper. The stark difference between the connotations of retractions and corrections is well reflected in their history and current prevalence: retractions are an extremely recent phenomenon that has grown in parallel with the strengthening of journal policies on misconduct, whilst corrections have been issued in all disciplines at constant rates for at least half a century and their frequency is still about 30 times higher than that of retractions [35].

What can corrections tell us about scientific integrity? Corrections are the consequence of a mistake, and might therefore reflect some degree of “sloppiness”. However, authors are not strictly obliged to correct their papers, and incur no punishment if they fail to do so. Corrections, in other words, are actions of little consequence, willingly carried out by researchers who wish to perfect their work, protect their reputation, and avoid misleading their colleagues. As such, corrections may be considered manifestations of scientific integrity. It follows that any sociological or psychological factor that increases the risk of scientific misconduct, and therefore the likelihood of retractions, should have, at a minimum, a smaller (null) effect on corrections, and possibly even an opposite effect.

This study verified whether the occurrence of a retraction or a correction could be predicted by study characteristics that reflect the six risk factors discussed above. Fifteen specific predictions were tested, all of which had been proposed explicitly or implicitly in the published literature. To avoid introducing subjectivity in the tests, we followed the classification schemes used in previous publications (Table 1). Sampling and analyses were designed to overcome limitations of past studies on retractions, including the skewing effects of prolific retractors [36] (see Methods). This study, therefore, is a direct and independent test of common beliefs and hypotheses about what might threaten scientific integrity.

**Table 1:** General hypothesised risk factors for research misconduct, parameters measured in this study, predicted association of such parameter with the likelihood to retract or correct, and summary of associations observed when selecting from each paper a multi-retracted author or, if this was not available, either the first or the last author (all numerical results are reported in S1 File).

factor	parameter measured	Predicted		Observed			
				Multi-r or first		Multi-r or last	
		ret	corr	ret	corr	ret	corr
**policies** [2–4]	country of author: legal RI structure (USA, DK, NO)	-/+ ‡	0+	-***	+	-**	0
	country of author: national RI policies (UK, SW, FI, NL, DE, AT, AU, JP, CN, KR, CR, TN, ZA)	-/+ ‡	0+	-	0	0	0
	country of author: local RI policies (ES, IL, FR, BE, CH, EE, LV, PL, CZ, HU, PE, GR, IN, BD)	-/+ ‡	0+	-***	0	-**	0
**culture** [6]	country of author: developmental state & German academia (CN, JP, KR)	+	0 -	0	0	0	0
	country of author: intermediate case (DE, SI, TW, ISR)	0	0	0	0	+*	0
	country of author: regulatory state & Anglo-American academia (US, UK)	-	0 +	-	0	-*	0
**pressures to publish** [11]	country of author: cash-incentives to individuals (CN, KR, TU)	+	0 -	0	0	+*	0
	country of author: performance linked to individual’s career (DE, ES, USA)	+	0 -	-	0	0	0
	country of author: performance linked to institution’s funding (AU, BE, NZ, DK, IT, NO, UK)	+	0 -	0	0	-*	0
	author's total number of papers, mean n. of papers per year	+	0 -	-	+	-***	+
	author's total citations, average citations per paper, av field-normalized citations, av field-normalized journal impact, proportion of papers in top 10% of relevant field(s)	+	0 -	-*	+***	-***	+***
**peer control** [7]	paper's number of co-authors	-	0 +	0	+***	0	+***
	paper's countries-to-author ratio	+	0 -	0	0	0	0
**early-career** [7,14]	author's number of years between first publication and: year of corrected/retracted paper, year of correction/retraction	-	0+	-**	0	-***	0
**Gender** [15,16]	given name of author: female vs. male vs. unknown	-	0 +	-	0	0	0

References indicate the source of the hypothesis tested and/or of the country policy classifications followed in this analysis. Countries are identified by their ISO codes. Zeroes amongst observed effects indicate any effect above the P>0.1 significance level, plus and minus signs alone, with one dot, and one, two or three asterisks indicate, respectively, effects at the P<0.1, P<0.05, P<0.01, and P<0.001 significance level, respectively (all numerical results are in the Supporting Information).

^‡^ predictions for this hypothesis are not straightforward: the presence of structures to deal with misconduct is predicted to decrease the likelihood to commit scientific misconduct, and therefore retractions; however, at least in the short term it should also increase the likelihood to uncover cases of misconduct, which could cause a rise in retractions.

## Methods

### General strategy

We collected a virtually complete set of bibliographic data on all co-authors of papers that have been retracted or corrected in 2010–2011 and compared them to control papers matched by journal and issue. Analyses were limited to one author per paper, and whenever possible we selected from each paper the author who, amongst his entire scientific production, had the largest number of retractions. Given that retractions are an extremely rare occurrence, this method identifies with high likelihood individuals who are responsible for scientific misconduct. For papers in which none of the authors had more than one retraction, we selected the first or the last author, positions that in most disciplines indicate the greatest involvement with the research, and examined each in a separate analysis. Moreover, this study is the first to avoid the biasing effect caused by “prolific retractors”, because it selected at random only one paper from each author that appeared multiple times in our sample. Combined with a conditional logistic regression analysis, this study design avoided the most important confounding factors that have limited past analyses, including differences in policies and practices across disciplines and within a journal over time. To ensure robustness of results, we further repeated all analyses taking simply the first, the last or a random author.

### Sample collection

The core of this study consisted in a large sample of errata and correction notes. This sample had been collected late in 2011 to conduct a descriptive study on errata and corrections that would represent as many research areas as possible. To this end, all records marked as “correction” or “correction, addition” were retrieved from the Web of Science (henceforth WOS) database, limiting the search to the years 2010 and 2011. The records were then partitioned by subject area (as defined in the WOS, and attributed based on journals) and, from each area that included a sufficient number of records, a random sample of 40 records (20 per each year) was drawn. Two research assistants (Leeanne Wood and Mark Tsun On Wong) hand-coded each correction and retrieved the WOS record corresponding to the corrected paper, which is cited by (and therefore linked to) the correction note. The current analyses used this list of corrected papers. The original sample of corrections included 279 additional corrections issued in the year 2000, which were excluded from the present study.

This sample included both *errata* (i.e. errors made and corrected by the journal editors, in which authors had no role) and actual corrections made by the authors. In order to ensure the inclusion only of the latter, analyses were repeated after excluding all corrections that had not been signed by all authors. Results were substantially similar to the results reported here, and are therefore omitted for brevity.

For each corrected and retracted paper in the database, we retrieved two control papers, published immediately before and after the retracted/corrected paper, in the same journal and issue. In cases where the retracted/corrected paper appeared at the end (or beginning) of a journal issue, we retrieved the paper published in the next (or previous) issue. When matched controls of different retracted/corrected papers overlapped, the next available paper, in either direction, was selected.

The retraction sample was retrieved in 2014, for the explicit purposes of this study, following methods of a previous analysis [35]. We collected all WOS records marked as “correction” or “correction, addition” that had “retraction” in the title and had been published in 2010 or 2011. Records were screened by hand and any record that was not a retraction note was removed. For all included retraction notes, the record of the retracted paper was retrieved through the cited reference that usually accompanies retraction notes. When more than one article was cited, the original paper was identified by matching its title to that of the retraction note.

The hand-coded characteristics of corrections were not used in this study, although they allowed us to identify several records that were marked as “corrections” in the WOS database even though they were in fact book reviews or similar items. These items, mostly found amongst papers in the humanities, were excluded from the sample. To avoid further spurious inclusions, we removed from all analyses any paper that was marked by the WOS database as: “Art Exhibit Review” (N = 4), “Bibliography” (N = 1), “Biographical-Item” (N = 24), “Book Review” (N = 159), “Dance Performance Review” (N = 1), “Film Review” (N = 69), “Music Performance Review” (N = 2), “News Item” (N = 37), “Note” (N = 1), “Poetry” (N = 2), “Record Review” (N = 1) and “Review; Book Chapter” (N = 1). Our results, however, were not sensitive to the inclusion/exclusion of these items.

The final sample of papers included in the analyses consisted in N = 611 retracted papers, N = 2226 corrected papers, and N = 1181 and N = 4285 matched controls, respectively. Depending on the specific analysis, these numbers are then subject to minor oscillations due to exclusion of duplicate author names (see below) or occasional missing information (sample size of each analysis is given in S1 File).

### Data collection

#### Basic study characteristics

Relevant bibliographic information was retrieved for all retraction and correction notes, for all the original retracted and corrected papers, and for all of their matched controls. In particular, we recorded information on:

- year of publication
- number of authors
- starting and ending page, from which the page length of the study was calculated
- names and surnames of all authors
- all addresses recorded. Countries specified in these addresses were used to calculate the countries-to-authors ratio, which is a proxy measure of the geographic distance amongst co-authors in each paper.

#### Gender of authors

The given names of all co-authors of all papers in our sample (N = 47,890) were retrieved, and gender was assigned using the given names of authors obtained from the WOS records. These given names were matched with a gender assignment table built from various census lists, webpages and wikis [37]. Not all journals, and therefore not all WOS records provide the full given names of authors. However, since the entire corpus of an author had been retrieved, we were able to assign given names to 42,156 authors (88% of the total), whilst 1210 names (2.5%) were classified as “unisex” and 4,524 (9.4%) had initials or unknown/unclassifiable names.

#### Country and bibliometric performance data of each author

The entire article production of each co-author (N = 47,890) of each paper included in the sample was identified using a disambiguation algorithm. This algorithm clusters WOS papers around individual author names, using decision rules that weight multiple items of information available in the WOS database records (e.g. first name, e-mail, affiliation etc.). Depending on the level of information available, the precision of this algorithm varies between 94.4% and 100%[38]. Following established methods [39], each corpus of literature associated with a disambiguated name in our sample was then used to calculate the following bibliometric parameters (named here as in the full-text):

- first publication year: year of the first publication of each author (as covered by WOS). This information was used to calculate two measures of career length:
  - Career length at publication: number of years occurring between an author’s first publication and the publication of the article that was included in our sample.
  - Career length at correction/retraction: number of years occurring between an author’s first publication and the year when the sampled paper was corrected or retracted.
- number of papers: total number of publications (article, review and letter) counted up to the year 2012.
- total citations: total number of citations (excluding author self-citations) for all the publications, considering citations received up to the year 2013.
- average citations: mean citation score of the publications calculated as the ratio of total citations to total number of publications.
- average normalized citations: mean field-normalized citation score of the publications. This is calculated by dividing each papers’ citations by the mean number of citations received from papers in the same WOS Subject Categories and year, and then by averaging these normalized scores across all papers of the author.
- average journal impact: mean field-normalized citation score of all journals in which authors have published. This measure would be conceptually similar to taking the average Journal Impact Factor of an author but, unlike the latter, it is not restricted to a two-year time window and is normalized by field.
- proportion top 10: proportion of publications of an author that belong to the top 10% most cited papers of their WOS Subject Categories.
- country of author: country was attributed based on the linkages between authors and affiliations recorded in all their papers available in the WOS. Since not all records have affiliation information, and since authors might report different affiliations throughout their careers, country was attributed based on a majority rule, taking into account all the countries associated with the author through publications. The country that we indicate, in other words, corresponds to the place of most (likely) frequent activity of the author. Due to lack of information, we could not attribute any country in this way to 3,345 authors (6.9% of the total). These cases were included in the “other country” category.

### Analyses

To approximate normality and/or to report regression effects on a unique scale, all continuous predictor variables except the country-to-author ratio were transformed as

$$log_{10}\left(1+x\right)$$

where *x* is the predictor of interest.

Measured characteristics of corrected or retracted papers were compared to those of their matched-control papers using conditional-logistic regression, which is the model best indicated for matched control study design on large samples. Given *j* groups consisting of corrected papers and matched-controls, if *p* is the probability of being a corrected paper rather than a control and *x* is the set of *n* author characteristics tested as predictors, the log-odds of a paper being corrected are given by:

$$\text{logit}\left(p\right)=\log\left(\frac{p}{1-p}\right)\;=\;\alpha +\alpha_{2}x_{s2}..+\alpha_{h}x_{sj}+\beta_{1}x_{1}+\ldots +\beta_{n}x_{n}$$ (1)

The conditional logistic regression model computes implicitly (“conditions out”) the first *j* terms on the right side of Eq 1, which correspond to the group-specific indicator variables. Estimates reported in all graphs, therefore, can be interpreted as the effects of study and author characteristics on the change in log-odds of being a retracted or corrected paper, adjusting for journal and issue—adjusting, in other words, for discipline, field, subfield, year and month of publication, journal retraction policy, mean journal impact and several other major confounding factors. Analyses were conducted using the package Survival, implemented in the open source statistical software R [40,41].

Effects of author characteristics on the number of retractions per author were instead analysed with a generalized linear model assuming Poisson distribution of errors and a quasi-likelihood function to account for over dispersion of data.

To assess the robustness of results, analyses were repeated identically using the following selection criteria for authors:

- *multi-retracted or first author*: if one or more co-authors of the included paper had more than one retracted paper amongst their entire literature production we selected the one with the highest number of retractions. If no co-author had more than one retraction, instead, we took the first author.
- *multi-retracted or last author*: same as above, but taking last author instead of first.
- *first author*: we always selected first author.
- *last author*: we always selected last author.
- *random author*: we selected one of the authors using a pseudo-random number generator

In each case, we avoided multiple inclusions of the same individual in the analysis (for example when the same individual was a first author in two or more papers included in the analysis) by selecting with a pseudo-random number generator one paper from that author and excluding the others.

### Disambiguation errors check

The algorithm that assigns papers to individual authors is efficient in proportion to available information on the author and how rare the name is. When information is scarce, certain categories of names (in particular, Asian or Hispanic names) run a higher risk of error in disambiguation, leading to multiple papers ascribed incorrectly to one author. To assess the possible effect of disambiguation errors on our results, we collected all names of authors to which the WOS database in 2012 had attributed unrealistically high numbers of papers, distinguishing three levels of implausibility: 730 records (i.e. the equivalent of having published two papers per day), 365 (one paper per day) and 183 (one paper every two days). Analyses were then repeated excluding authors that had any of these names from the sample. Having observed no substantial change in the overall results, we omitted these analyses from the text for brevity.

## Results and Discussion

Table 1 summarizes the general hypotheses tested, parameters measured, effects predicted and results obtained by analysing data on multi-retracted and first authors (henceforth, MRF) as well as multi-retracted and last authors (MRL, see Methods for explanations). In this section section we illustrate and discuss primarily results obtained on MRL, and will refer to other analyses only when needed (numerical results for all analyses are reported in the Supporting Information). Compared to MRF, results obtained on MRL showed a greater number of statistically significant effects, although in most cases the direction of observed effects was the same (Table 1, S1 File). The higher statistical significances observed with last authors are likely to be explained by the higher precision and statistical power of these analyses, achieved because last authors tend to be older and more highly ranked than first authors [42] and have published on average a larger number of papers (mean±SD = 29±66 and 75±112, respectively). An alternative interpretation, perhaps applicable to a subset of parameters and worth exploring in future research, is that these differences reflect genuine behavioural asymmetries between last and first authors.

**Fig 1:**
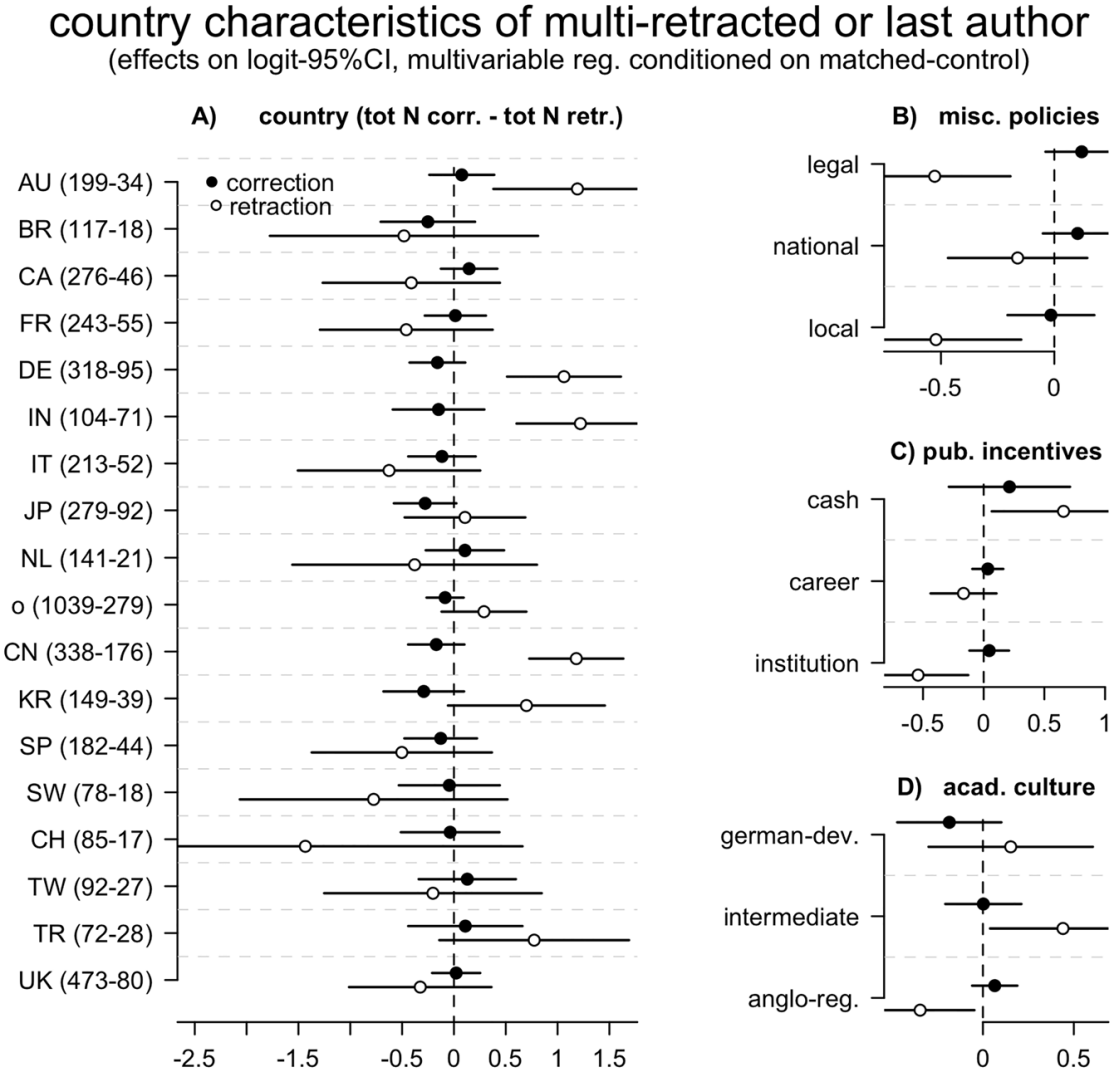
Retraction and correction likelihood, by country characteristics. Conditional logistic regression estimates of the association between country of author and likelihood to publish a paper that was later retracted or corrected. Effects are estimated by comparison with matched–control papers. Numbers in parentheses indicate the total number of papers from the specified country that are in the sample, respectively, of corrections and retractions. Each panel represents the results of two multivariable analyses, in which samples for correction and for retraction were analysed using identical models. The indicator reference category was USA (N: 1979 – 449) for panel A, and a generic “other countries” category in all other panels. The “other” category in panel A includes all countries with ≤90 data points in the sample. See Table 1 and Methods for further details.

The country of activity of an author (see methods) was a highly significant predictor of retraction. Australia, Germany, China South Korea and Turkey were more likely to host authors of retracted papers compared to the United States and, particularly in the case of MRF, The Netherlands and France were less likely. Countries differed much less in their correction likelihood, and effects were not statistically significant in most main and sensitivity analyses (Fig 1a, S1a Fig).

The between-country variance in retraction rates was significantly explained by national policies, but not always in the direction predicted. The likelihood of retraction was lower in countries that have policies and structures to handle allegations of misconduct, particularly when such policies are legally defined or institutional (Fig 1b, S1b Fig). The likelihood of a retraction was higher in countries where publication performance is rewarded with cash, in agreement with predictions. Contrary to predictions, however, the likelihood of a retraction was equal or lower in countries in which publication performance determines individual careers or national funding to institutions (Fig 1c, S1c Fig). Since true “pressures” are supposed to occur in these two latter categories and not in the former, these results contradict the pressures to publish hypothesis.

None of the policy categories considered was a significant predictor of corrections, consistent with our assumption that corrections are a spontaneous action that is felt to be inconsequential and is therefore indifferent to policies. Moreover, there was no overlap, in our sample, between authors of retracted and corrected papers, adding further credit to our assumption that corrections and retractions are distinct phenomena.

Countries with a regulatory structure and Anglo-American academic model—i.e. a model believed to optimize institutional control and peer criticism [6]—were significantly less likely to yield retractions, whilst culturally intermediate cases were most likely. German-developmental countries, where the risk of misconduct is predicted to be greatest, exhibited a non-significant tendency to produce more retractions and fewer corrections (Fig 1d S1d Fig). Again, no significant effect was observed for corrections, although the order in which effect sizes are distributed matches predictions almost linearly. Overall, therefore, these trends are in good agreement with the cultural hypothesis (Table 1).

**Fig 2:**
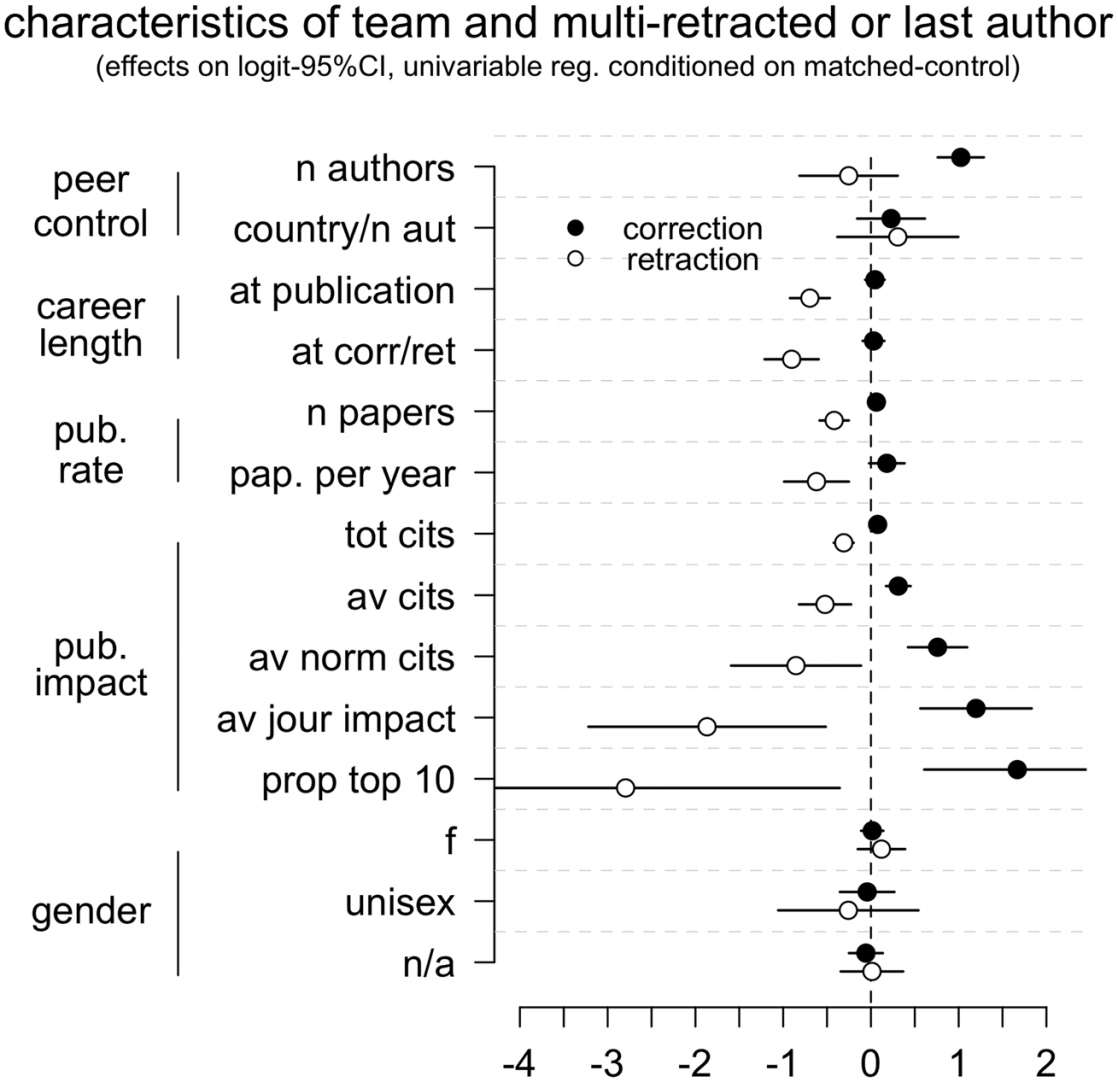
Retraction and correction likelihood, by team and individual characteristics. Conditional logistic regression estimates of the association between author or team characteristics and likelihood to publish a paper that was later retracted or corrected. Effects are estimated by comparison with matched-control papers. Corrections and retractions were analysed separately using identical univariable analyses, testing each parameter in turn. The gender was analysed in a multivariable model, in which “male” was the reference category. All predictors except gender were log-transformed. Parameters are grouped by the general risk factor of which they are proxies. For further details, see Table 1 and Methods.

A paper’s number of co-authors increased the likelihood of it being later corrected, but was not a significant predictor of it being retracted, yielding a partial support for the peer-control hypothesis. The countries-to-authors ratio was not associated with corrections or retractions (Fig 2, S2 Fig). This latter finding represents a null support for the peer-control model, but may not be considered a strong refutation, since the proxy used to measure the distance between authors was rather inaccurate.

As predicted by the early-career hypothesis, researchers who had a shorter publication history (measured twice, at the time of publishing the original paper and at the time of correcting/retracting it) were more likely to be the authors of a retracted paper, whilst no effect was observed for corrections (Fig 2, S2 Fig).

Against all predictions derived from the pressures to publish hypothesis, retracted papers were less likely, and corrected papers more likely to be authored by researchers that were highly productive and had published frequently in high-impact journals (Fig 2, S2 Fig). Effect sizes were larger, but with wider margins of error, for the size-independent and field-independent measurements (i.e. total number citations showed smaller effects than average citations per paper, which in turn showed smaller effects than average and normalized citations) suggesting the presence of a genuine effect underlying all these measures (Fig 2, S2 Fig).

The sex of the author’s first name was not significantly associated with the likelihood of either retraction or correction, in any of the analyses conducted (Fig 2, S2 Fig, S1 File). Retractions showed a non-statistically significant tendency to be authored less by female first authors, and equally or more by female last authors. These patterns might suggest that a link between gender and misconduct is modulated by career or status. Original speculations on the role of gender in scientific misconduct also suggested a career/status effect, but one that was opposite to the effect noted here [15]. Therefore, whilst these results may not rule out entirely a gender effect, they offer a null or a negative support for the gender hypothesis as formulated in the literature. Future studies should explore in greater detail the link between gender, academic status and scientific misconduct.

Overall, our findings support previous suggestions that national policies, socio-cultural conditions, research environment (including number of co-authors) and situational factors (i.e. career stage) are significant determinants of responsible and irresponsible practices. Effect sizes are in the medium to large range (see S1 File, the “exp(coef)” values correspond to change in odds ratio), which suggests that these risk factors have practical, not just statistical, significance. The hypothesis that males are prone to scientific misconduct, however, was not supported, and the widely held belief that pressures to publish are a major threat to scientific integrity was largely contradicted by analyses at the level of country as well as individual (Table 1).

**Fig 3:**
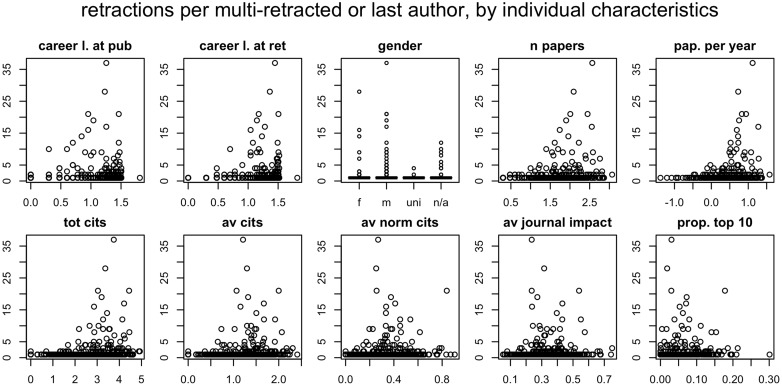
Number of retractions per author, by team and individual characteristics. Total number of retracted papers attributed, in the WOS, to authors included in this study (y axis), plotted against various individual performance parameters (x-axis). All predictor parameters were log-transformed. Authors with no retractions were omitted. For further details, see Fig 2 and Methods.

Perceived problems with pressures to publish might be just a “shared myth” [43]. But, if that is the case, why is the myth so widely believed? Part of the explanation might lie in the disproportionate attention paid to extreme cases of fraud. Within our sample, we observed that extreme cases of “prolific retractors” (authors of several retracted papers) tended to fall in the highest percentiles of productivity, even though their average journal impact was mediocre (Fig 3). Since these cases often represent spectacular examples of fraud, they tend to attract the attention of the scientific community and the mass media, and thus might contribute to reinforcing a stereotype. Interestingly, these authors also tended to be male, so these extreme cases might reinforce gender stereotypes, too (Fig 3).

**Fig 4:**
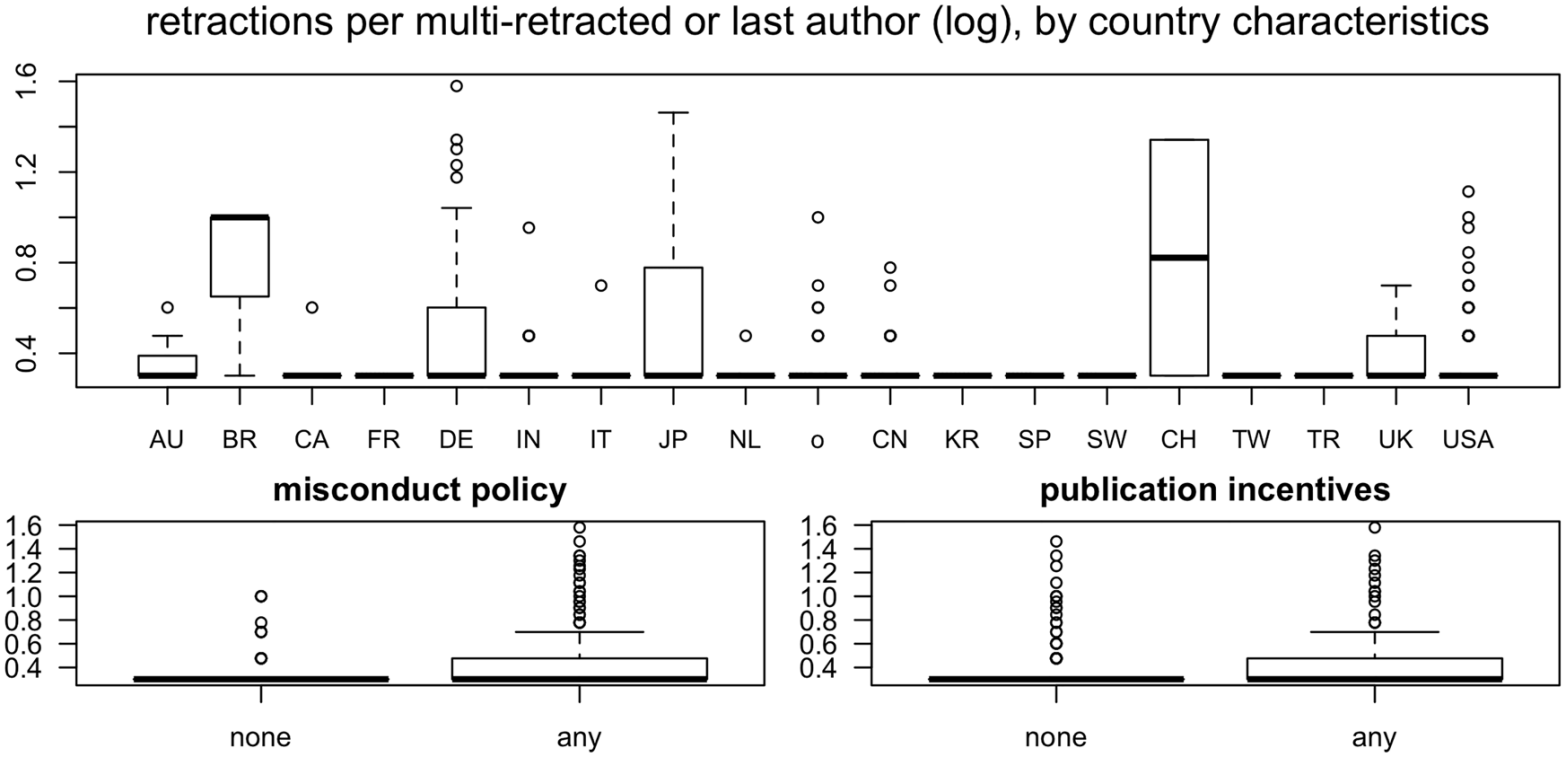
Number of retractions per author, by country characteristics. Total number of retracted papers attributed, in the WOS, to authors included in this study, by country of activity (top) and by policy characteristics of those countries (bottom). For further details, see Table 1 and Methods.

Just like the growth in retractions is explained by changes in journal policies [29], the rise of cases of prolific retractors can be explained by the strengthening of national policies against scientific misconduct. Authors from countries that have misconduct policies of any kind had a higher average number of retractions per individual (they were, in other words, more likely to be “prolific retractors”), as would be expected if multiple retractions by one individual followed from thorough investigations. Authors from countries in which performance was incentivized, instead, did not have significantly more retractions per individual, as would have been predicted by the pressures to publish scenario (Fig 4, see S1 File for results on first authors). This analysis, however, was conducted *post hoc* and is very coarse-grained (see Limitations section). We cannot exclude that extreme cases of prolific retractors might result from stronger incentives or pressures to publish, and future research should test this hypothesis.

The fundamental conclusions drawn from this study are supported by multiple sensitivity analyses. Effects observed in most parameters were similar in direction or null, but never opposite, when analyses were repeated using multivariable regression (S3 and S4 Figs), in analyses limited to US authors (S4 and S6 Figs) and in tests using different author selection criteria (S1 File). Moreover, results were not significantly affected by name disambiguation errors (see Methods). Our inclusion criteria for corrections were conservative because they included corrections that had not been signed by the authors of the original paper (which might represent *errata*, i.e. corrections of editorial mistakes). If these non-author-signed correction were excluded (sample size: N = 1054 corrections and N = 2035 matched controls), results were substantially unchanged. Inclusion criteria for retractions were also conservative, because they included retractions due to honest mistakes. We tried limiting analyses to retractions that had not been signed by all authors (which might indicate a serious disagreement and therefore a possible case of misconduct, N = 556) and results obtained were not substantially different. However, if assumptions underlying this study are correct, then retractions due to honest errors should exhibit patterns similar to those observed for corrections and different or opposite to those observed for retractions. This is a new prediction that future studies should test.

### Limitations

This study did not distinguish between retractions due to data fabrication/falsification and those due to plagiarism. This is in theory a relevant limitation because, although the motivation underlying all these forms of scientific misconduct is ultimately the same (i.e. to gain an unfair advantage in the race for priority and success [35]), their phenomenology might be different. Plagiarists, for example, might be especially motivated to seek low-impact journals in order to escape scrutiny. The broad confidence intervals observed for some effects, and in particular parameters measuring a researcher’s impact (Fig 2, S2 Fig) are compatible with the hypothesis that some forms of misconduct are more susceptible to these effects than others. However, since confidence intervals overlapped modestly or not at all with zero, it is unlikely that our results conceal *opposite* effects. It is unlikely, in other words, that either fabrication/falsification or plagiarism are *more* common amongst researchers that publish more frequently and in higher-impact journals, which are the only findings that would support the pressures to publish scenario and contradict this study’s conclusions. Future research should nonetheless test for differential effects amongst categories of scientific misconduct.

Our study design removed several limitations of past analyses. Nonetheless, our results consist in retrospective observational data about an extremely complex phenomenon. Our findings are, therefore, unavoidably conditioned to auxiliary hypotheses, open to alternative explanations, and unable to conclusively prove cause-effect relationships. The various country categories that we have tested overlap with one another and there are simply not enough countries within these categories to distinguish the effects of policies from those of publication incentives or culture. Characteristics of individual researchers, moreover, are not independent of the environment in which they operate. We were able to show that when analyses are restricted to one country, i.e. USA, effects observed are similar or null, but not opposite to those observed across countries (S4 Fig). However, we were unable to control for lower-level effects. Therefore, we cannot exclude that highly productive and high-impact researchers might be operating in research institutions in which greater attention is paid to scientific integrity and in which rules for scientific misconduct are applied more rigorously. Similarly, early career researchers might be proportionally more abundant in areas of the world where policies or cultures are unfavourable to scientific integrity. Additionally, due to their low academic status, early-career researchers could be more vulnerable to allegations of scientific misconduct and less able to defend themselves. Early-career researchers might also be subject to the strongest pressures to publish. Against the latter hypothesis, however, we did not observe a significant interaction effect, on retractions, of career stage and performance parameters. This suggests that pressures to publish, independent of career stage, might at best represent a psychological risk factor, with highly subjective effects.

### Conclusions

Albeit observational, our results clearly contradict some, and support other beliefs about which factors are threatening most severely scientific integrity (Table 1), and thus bear multiple theoretical and practical implications. First, whilst cash incentives might have detrimental effects on scientific integrity, as commonly suspected, other forms of performance stimulation cannot be said to increase the risk of scientific misconduct, and may have null or even positive effects. Second, since productive, high-impact scientists exhibit above-average integrity, the “bulk of the iceberg” of scientific misconduct is likely to be found amongst low-profile journals and mediocre authors. Third, countries that still lack structures and policies to handle allegations of scientific misconduct are characterized by a higher risk to produce fraudulent papers, and are therefore most urgently in need of regulations. Indeed, our findings support recent evidence suggesting that scientific misconduct might be very high in developing countries [44,45]. Fourth, younger researchers and those working in situations in which mutual criticism is hampered might be at greater risk of engaging in scientific misconduct.

In conclusion, our results suggest that policies to reduce pressures to publish might be, as currently conceived, ineffective, whereas establishing policies and structures to handle allegations of scientific misconduct, promoting transparency and mutual criticism between colleagues, and bolstering training and mentoring of young researchers might best protect the integrity of future science.

## Supporting Information

S1 Fig Retraction and correction likelihood, by country characteristics. Conditional logistic regression estimates of the association between country of author and likelihood to publish a paper that was later retracted or corrected. Effects are estimated by comparison with matched-control papers. Numbers in parentheses indicate the total sample sizes (experimental + controls) for corrections and retractions. Each panel represents the results of two multivariable analyses, in which samples for correction and for retraction were analysed using identical models. The indicator reference category was USA (N: 2789 – 1561) for panel A, and a generic “other countries” category in all other panels. The “other” category in panel A includes all countries with ≤90 data points in the sample. See Table 1 and Methods for further details.
(TIF)

S2 Fig Retraction and correction likelihood, by team and individual characteristics. Conditional logistic regression estimates of the association between author or team characteristics and likelihood to publish a paper that was later retracted or corrected. Effects are estimated by comparison with matched-control papers. Corrections and retractions were analysed separately using identical univariable analyses, testing each parameter in turn. The gender was analysed in a multivariable model, in which “male” was the reference category. All predictors except gender were log-transformed. Parameters are grouped by the general risk factor of which they are proxies. For further details, see Table 1 and Methods.
(TIF)

S3 Fig Retraction and correction likelihood, by team, individual and country characteristics. Multiple conditional logistic regression estimates of the association between study characteristics and likelihood to be later retracted or corrected. Corrections and retractions were analysed separately, in identical multivariable models. Effects are estimated by comparison with matched control papers. All continuous predictors except country/author ratio were log-transformed. The length of the article (number of pages) was included in this model because it is a relevant confounding factor. The reference category for the country variable was USA. For further details, see Table 1 and Methods.
(TIF)

S4 Fig Retraction and correction likelihood, by team, individual and country characteristics. Multiple conditional logistic regression estimates of the association between study characteristics and likelihood to be later retracted or corrected. Corrections and retractions were analysed separately, in identical multivariable models. Effects are estimated by comparison with matched control papers. All continuous predictors except country/author ratio were log-transformed. The length of the article (number of pages) was included in this model because it is a relevant confounding factor. The reference category for the country variable was USA. For further details, see Table 1 and Methods.
(TIF)

S5 Fig Retraction and correction likelihood, by team and individual characteristics. Univariable conditional logistic regression estimates of the association between author or team characteristics and likelihood to publish a paper that was later retracted or corrected (see Table 1 for further details), with analyses limited to authors working in the United States. Effects are estimated by comparison with matched-control papers. Corrections and retractions were analysed separately using identical univariable analyses, testing each parameter in turn. The gender was analysed in a multivariable model, in which “male” was the reference category. All predictors except gender were log-transformed. Parameters are grouped by the general risk factor of which they are proxies. For further details, see Table 1 and Methods
(TIF)

S6 Fig Retraction and correction likelihood, by team and individual characteristics. Univariable conditional logistic regression estimates of the association between author or team characteristics and likelihood to publish a paper that was later retracted or corrected (see Table 1 for further details), with analyses limited to authors working in the United States. Effects are estimated by comparison with matched-control papers. Corrections and retractions were analysed separately using identical univariable analyses, testing each parameter in turn. The gender was analysed in a multivariable model, in which “male” was the reference category. All predictors except gender were log-transformed. Parameters are grouped by the general risk factor of which they are proxies. For further details, see Table 1 and Methods.
(TIF)

S1 File Numerical results of all analyses. The file contains the R output of all analyses conducted, including those underlying the figures reported in the text as well as robustness analyses as described in the Materials and Methods section.
(TXT)

S2 File Data file. Data sets used in all analyses and figures. Paper identifiers and author identifiers and names have been anonymized due to restrictions that apply to bibliometric data.
(TXT)
