# Supplementary figures and images for: Misconduct Policies, Academic Culture and Career Stage, Not Gender or Pressures to Publish, Affect Scientific Integrity
Source: PLoS One. 2015 Jun 17;10(6):e0127556. doi: 10.1371/journal.pone.0127556 (PMC4471332; doi:10.1371/journal.pone.0127556)

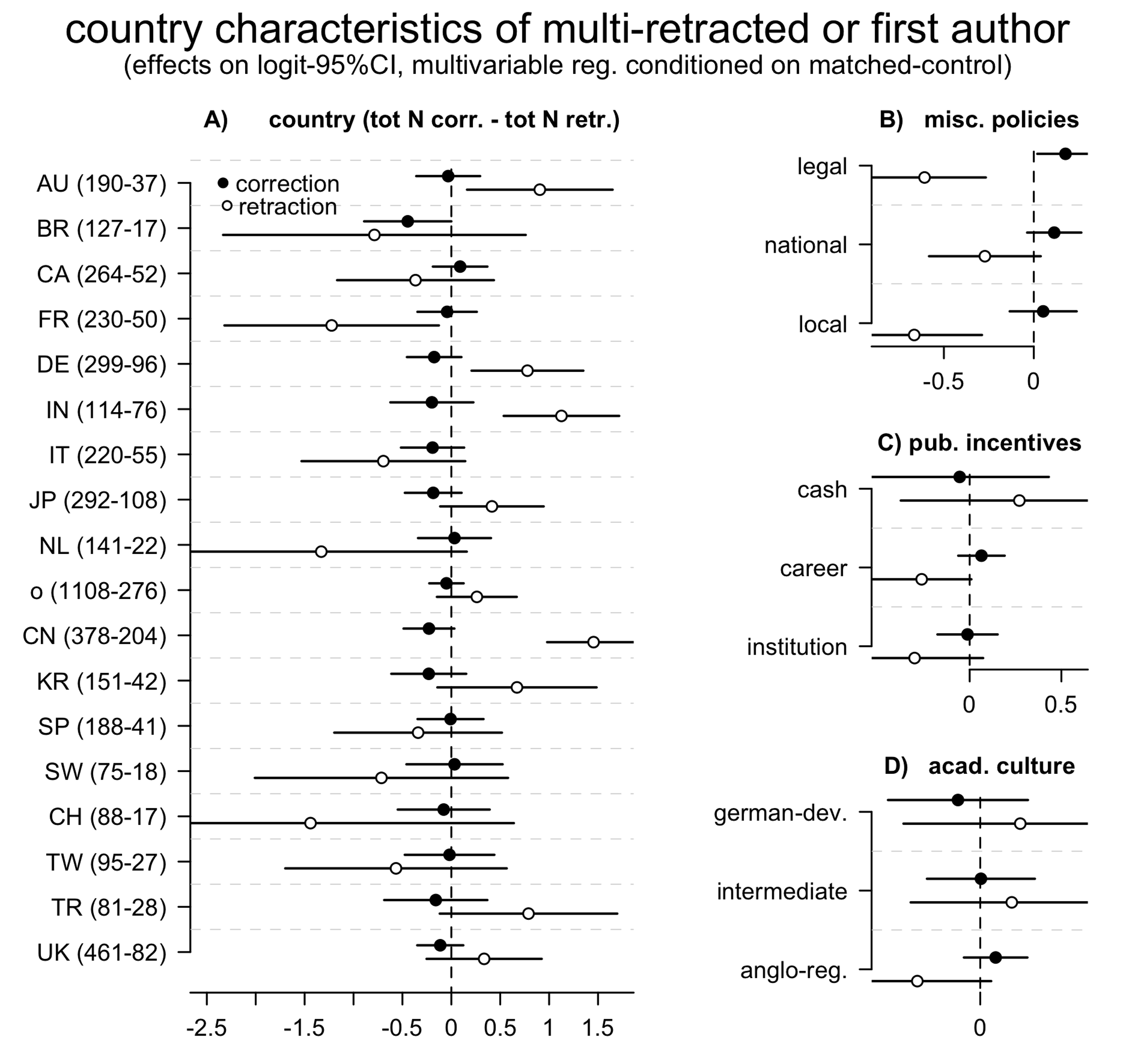

Supplement: S1 Fig — Conditional logistic regression estimates of the association between country of author and likelihood to publish a paper that was later retracted or corrected. Effects are estimated by comparison with matched-control papers. Numbers in parentheses indicate the total sample sizes (experimental + controls) for corrections and retractions. Each panel represents the results of two multivariable analyses, in which samples for correction and for retraction were analysed using identical models. The indicator reference category was USA (N: 2789 – 1561) for panel A, and a generic “other countries” category in all other panels. The “other” category in panel A includes all countries with ≤90 data points in the sample. See Table 1 and Methods for further details. (TIF) [file pone.0127556.s001.tif]

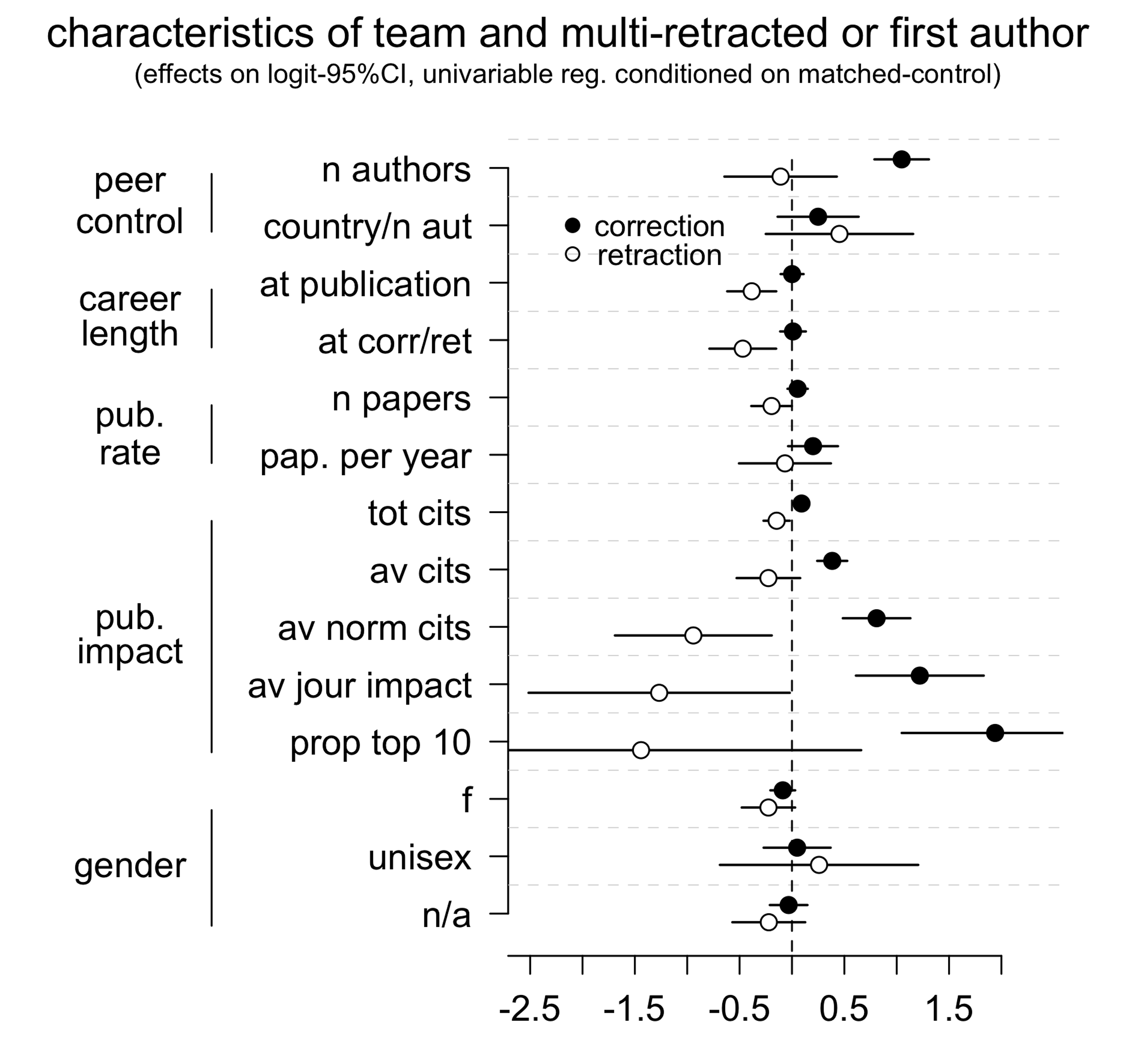

Supplement: S2 Fig — Conditional logistic regression estimates of the association between author or team characteristics and likelihood to publish a paper that was later retracted or corrected. Effects are estimated by comparison with matched-control papers. Corrections and retractions were analysed separately using identical univariable analyses, testing each parameter in turn. The gender was analysed in a multivariable model, in which “male” was the reference category. All predictors except gender were log-transformed. Parameters are grouped by the general risk factor of which they are proxies. For further details, see Table 1 and Methods. (TIF) [file pone.0127556.s002.tif]

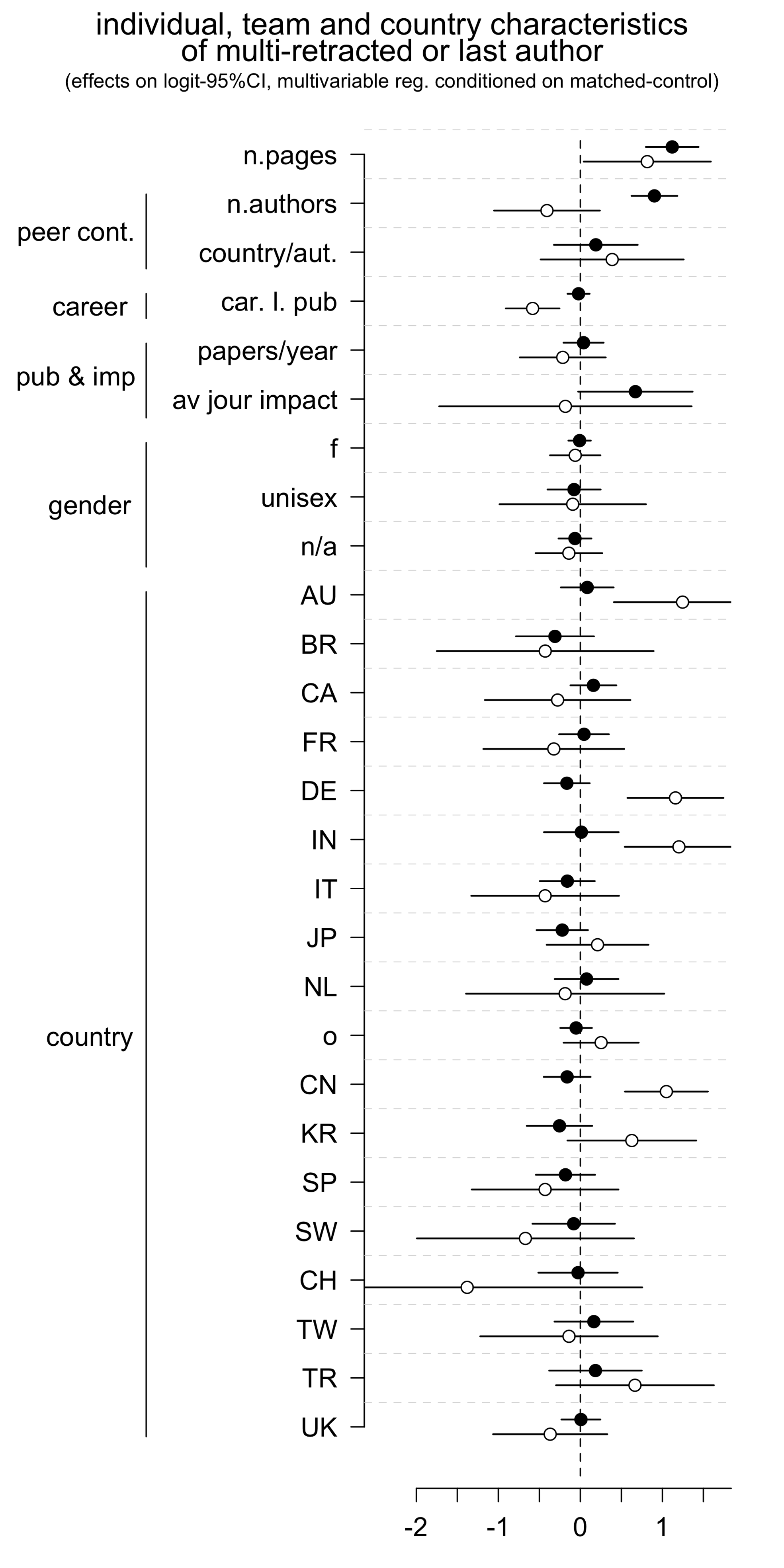

Supplement: S3 Fig — Multiple conditional logistic regression estimates of the association between study characteristics and likelihood to be later retracted or corrected. Corrections and retractions were analysed separately, in identical multivariable models. Effects are estimated by comparison with matched control papers. All continuous predictors except country/author ratio were log-transformed. The length of the article (number of pages) was included in this model because it is a relevant confounding factor. The reference category for the country variable was USA. For further details, see Table 1 and Methods. (TIF) [file pone.0127556.s003.tif]

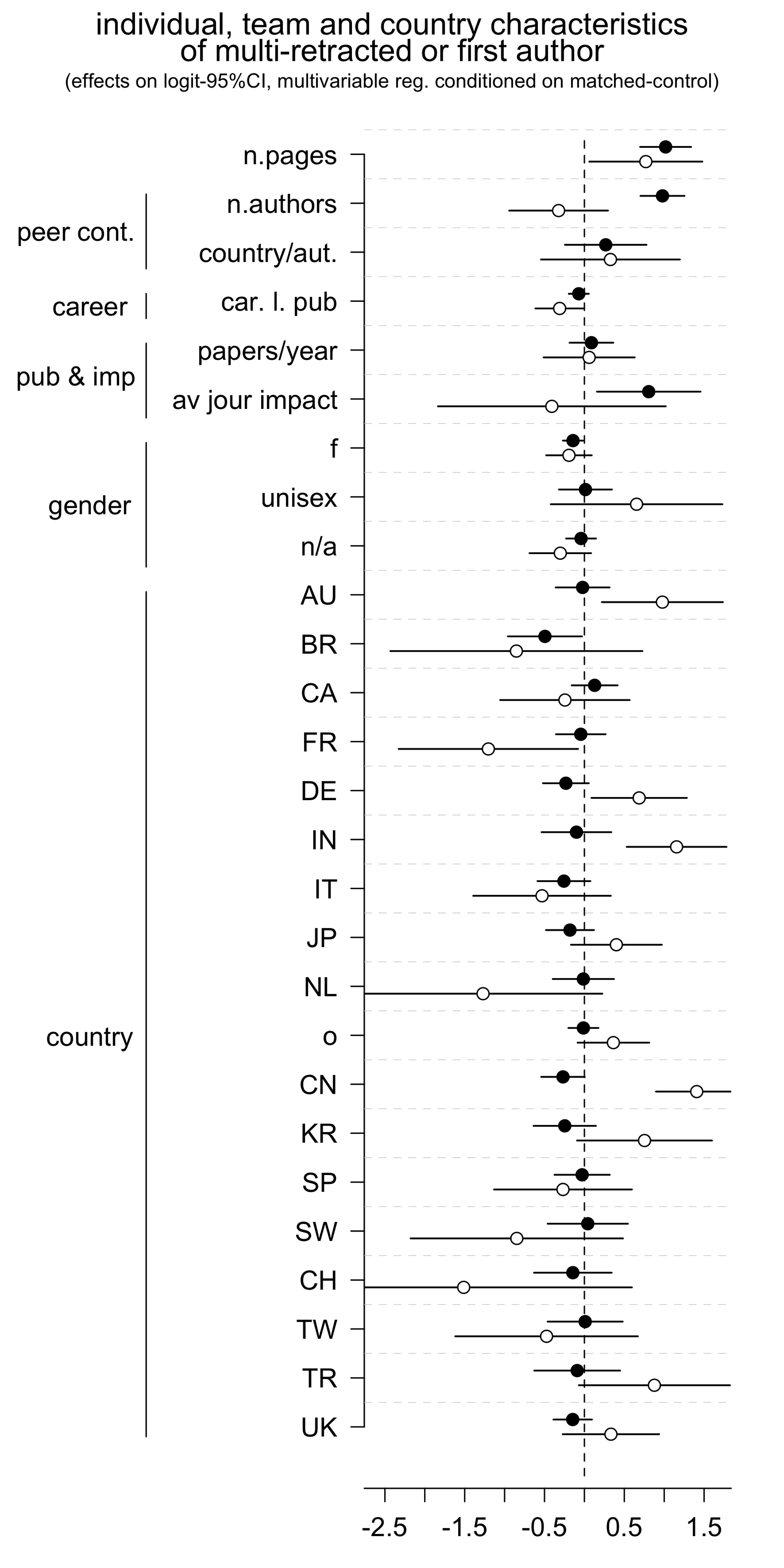

Supplement: S4 Fig — Multiple conditional logistic regression estimates of the association between study characteristics and likelihood to be later retracted or corrected. Corrections and retractions were analysed separately, in identical multivariable models. Effects are estimated by comparison with matched control papers. All continuous predictors except country/author ratio were log-transformed. The length of the article (number of pages) was included in this model because it is a relevant confounding factor. The reference category for the country variable was USA. For further details, see Table 1 and Methods. (TIF) [file pone.0127556.s004.tif]

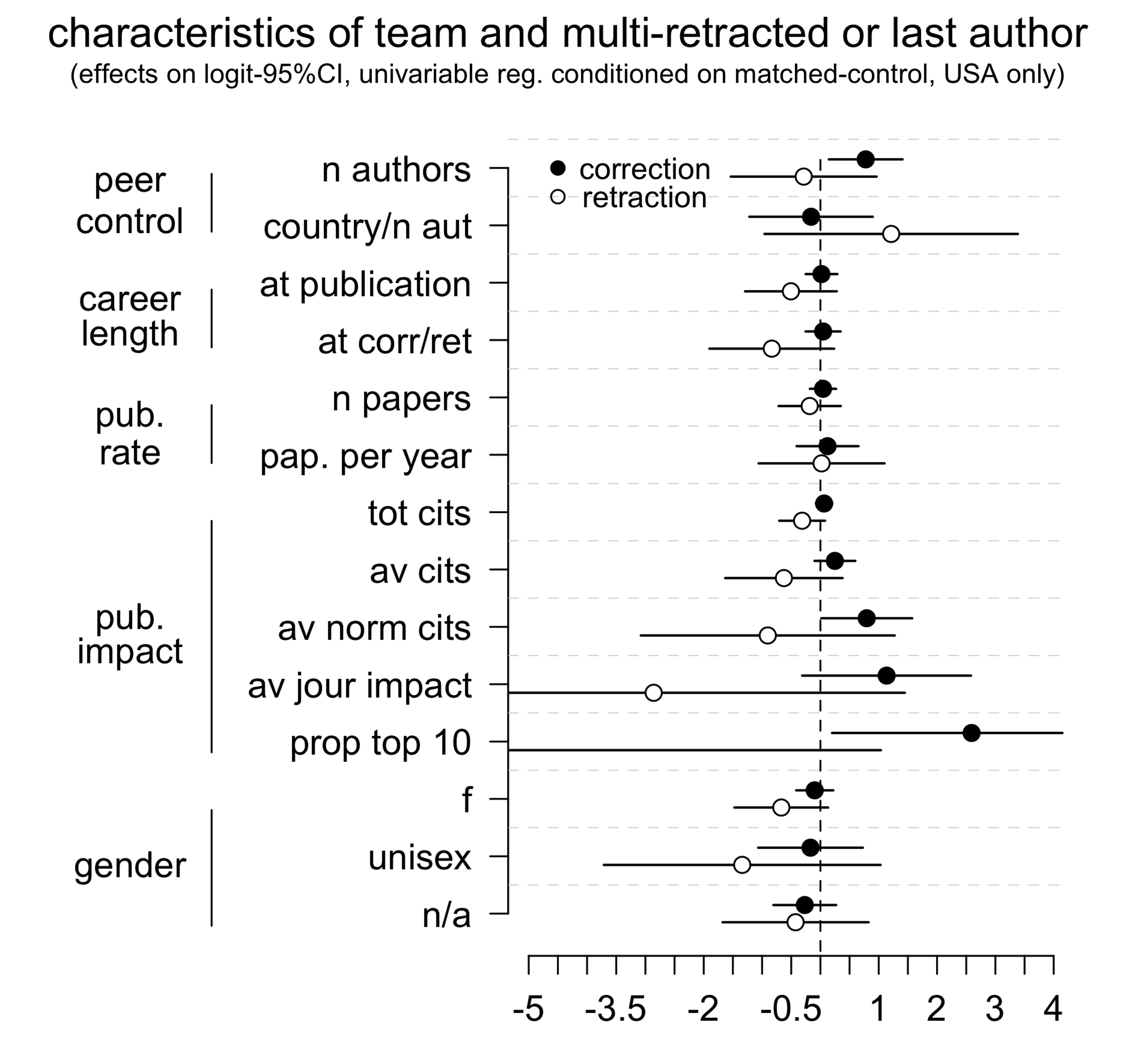

Supplement: S5 Fig — Univariable conditional logistic regression estimates of the association between author or team characteristics and likelihood to publish a paper that was later retracted or corrected (see Table 1 for further details), with analyses limited to authors working in the United States. Effects are estimated by comparison with matched-control papers. Corrections and retractions were analysed separately using identical univariable analyses, testing each parameter in turn. The gender was analysed in a multivariable model, in which “male” was the reference category. All predictors except gender were log-transformed. Parameters are grouped by the general risk factor of which they are proxies. For further details, see Table 1 and Methods (TIF) [file pone.0127556.s005.tif]

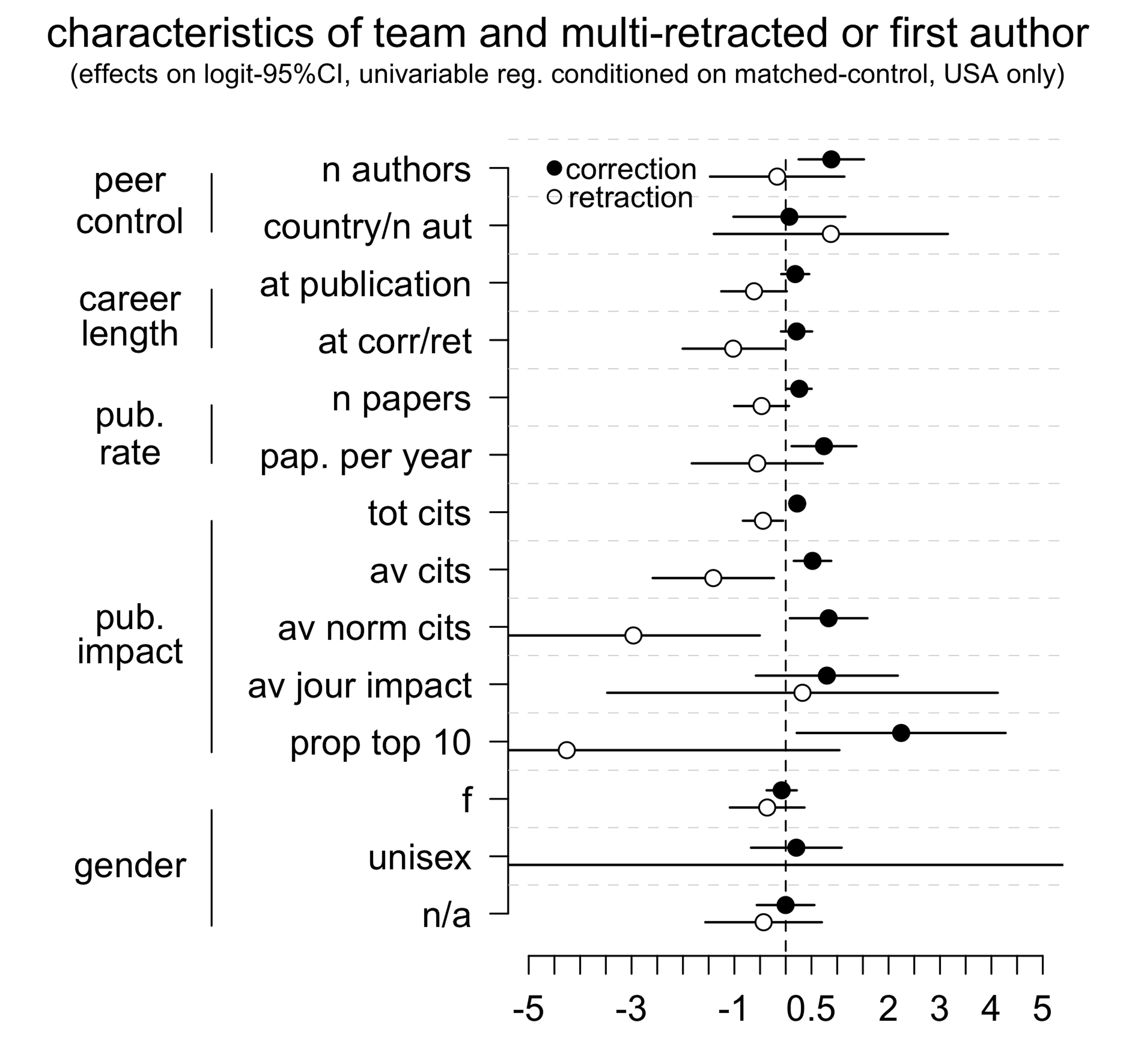

Supplement: S6 Fig — Univariable conditional logistic regression estimates of the association between author or team characteristics and likelihood to publish a paper that was later retracted or corrected (see Table 1 for further details), with analyses limited to authors working in the United States. Effects are estimated by comparison with matched-control papers. Corrections and retractions were analysed separately using identical univariable analyses, testing each parameter in turn. The gender was analysed in a multivariable model, in which “male” was the reference category. All predictors except gender were log-transformed. Parameters are grouped by the general risk factor of which they are proxies. For further details, see Table 1 and Methods. (TIF) [file pone.0127556.s006.tif]
